# Enhanced Growth Performance and Salinity Tolerance in Transgenic Switchgrass via Overexpressing Vacuolar Na^+^ (K^+^)/H^+^ Antiporter Gene (*PvNHX1*)

**DOI:** 10.3389/fpls.2017.00458

**Published:** 2017-04-03

**Authors:** Yanhua Huang, Cong Guan, Yanrong Liu, Baoyue Chen, Shan Yuan, Xin Cui, Yunwei Zhang, Fuyu Yang

**Affiliations:** ^1^Department of Crop Ecology and Farming, College of Agriculture and Biotechnology, China Agricultural UniversityBeijing, China; ^2^Department of Grassland Science, College of Animal Science and Technology, China Agricultural UniversityBeijing, China; ^3^Beijing Key Laboratory for Grassland Science, China Agricultural UniversityBeijing, China; ^4^National Energy R&D Center for BiomassBeijing, China; ^5^Beijing Sure Academy of BiosciencesBeijing, China

**Keywords:** switchgrass, vacuolar Na^+^ (K^+^)/H^+^ antiporter, *PvNHX1*, growth, salt tolerance

## Abstract

Switchgrass (*Panicum virgatum* L.) has been increasingly recognized as one of the most valuable perennial bioenergy crop. To improve its biomass production, especially under salt stress, we isolated a putative vacuolar Na^+^ (K^+^)/H^+^ antiporter gene from switchgrass and designated as *PvNHX1*. Subcellular localization revealed that this protein was localized mainly on the vacuole membrane. The *PvNHX1* was found to be expressed throughout the entire growth period of switchgrass, exhibited preferentially expressed in the leaf tissue, and highly induced by salt stress. Transgenic switchgrass overexpressing *PvNHX1* showed obvious advantages with respect to plant height and leaf development compared to the wild-type (WT) and transgenic control (EV, expressing the empty vector only) plants, suggesting PvNHX1 may serve as a promoter in switchgrass growth and development. Moreover, transgenic switchgrass were more tolerant than control plants with better growth-related phenotypes (higher shoot height, larger stem diameter, longer leaf length, and width) and physiological capacities (increased proline accumulation, reduced malondialdehyde production, preserved cell membrane integrity, etc.) under high salinity stress. Furthermore, the genes related to cell growth, flowering, and potassium transporters in transgenic switchgrass exhibited a different expression profiles when compared to the control plants, indicating a pivotal function of PvNHX1 in cell expansion and K^+^ homeostasis. Taken together, PvNHX1 is essential for normal plant growth and development, and play an important role in the response to salt stress by improving K^+^ accumulation. Our data provide a valuable foundation for further researches on the molecular mechanism and physiological roles of NHXs in plants.

## Introduction

Plants constantly face threats from diverse biotic and abiotic factors, and salinity stress has become one of the major abiotic factors which adversely affects the plant growth, sustainability and productivity, even drove dual consequences of osmotic stresses and specific ion toxicities. Through adaptation, plants also have developed efficient strategies to survive in high salinity conditions with an array of morphological, physiological, biochemical, and molecular mechanisms (Krasensky and Jonak, 2012). The maintenance of cellular pH and ion homeostasis, especially intracellular K^+^ and Na^+^, is an important strategy to ensure optimal conditions for plant growth and development under high salt stress conditions among these mechanisms (Reguera et al., 2014).

Intracellular Na^+^ (K^+^)/H^+^ antiporters (NHXs) belong to the large family of monovalent cation/H+ antiporters CPA1, which contribute to cellular pH and Na^+^, K^+^ homeostasis in plants (Chanroj et al., 2012). NHXs catalyze the electroneutral exchange of Na^+^ and/or K^+^ for H^+^ using the electrochemical H^+^ gradients generated by vacuolar H^+^-translocating enzymes, H^+^-adenosine triphosphatase (ATPase) and H^+^-inorganic pyrophosphatase (H^+^-PPiase) to maintain both luminal pH, as well as the intracellular cation homeostasis (Bassil and Blumwald, 2014).

NHXs are ubiquitous to all eukaryotic organisms and implicate a pivotal function in regulating plant responses to salt stress (Zhang et al., 2015; Sahoo et al., 2016), cold tolerance (Li et al., 2007), drought tolerance (Wang et al., 2016) and disease resistance (Chen et al., 2015). The successful expressions of the key enzyme genes related to the *NHXs* were proved to be valid to enhance the capacity for salt tolerance in diverse species, such as and cowpea (Mishra et al., 2014), tobacco (Chen et al., 2015), alfalfa (Zhang et al., 2015), and mungbean (Sahoo et al., 2016). In spite of numerous work has focused on use of NHXs in salt tolerance, the mechanism underlying the enhancement of salinity tolerance by NHXs remained unclear (Sahoo et al., 2016; Wang et al., 2016). In plants, the sequestration of Na^+^ in vacuole had been identified as an important mechanism to salt tolerance (Apse et al., 1999). However, recent studies have found the opposite result in transgenic rice (Islam et al., 2010), soybean (Li et al., 2010), and cowpea (Mishra et al., 2014), that greater K^+^, rather than Na^+^ contents were observed under salinity stress. Emerging new reverse genetics evidence has also implicated that NHXs were not indispensable for Na^+^ uptake into vacuoles of *Arabidopsis*, and confirmed their main role in the regulation of K^+^ homeostasis (Bassil et al., 2011; Barragán et al., 2012; Andrés et al., 2014). Furthermore, NHXs were also involved in cellular metabolism, plants growth, flowering and reproduction by regulating cellular pH and K^+^ homeostasis (Yoshida et al., 2009; Bassil et al., 2011; Barragán et al., 2012).

Switchgrass (*Panicum virgatum* L.), a perennial C4 warm-season grass belonging to the *Poaceae*, has been increasingly recognized as a dedicated bioenergy crop for its agricultural, industrial, and ecological advantages (Brown et al., 2015). Nowadays the cultivations within the marginal land are concerned, screening varieties of high resistance especially the salt resistance will be of great significance. However, as an outcrossing and polyploid species, conventional strategies were severely challenged in switchgrass new varieties breeding processes (Xu et al., 2011). Direct introduction of salt tolerance related genes by genetic engineering is expected to be highly efficient in generating novel varieties of switchgrass with improved biomass on salinity affected areas. The pivotal function of NHXs in response to salt stress has been proposed in diverse plant species, whereas have not been confirmed yet in switchgrass, the most promising model energy grass for cellulose ethanol production.

In the present study, we isolated a Na^+^ (K^+^)/H^+^ antiporter gene from switchgrass and analyzed its subcellular localization in plant cells by transient expression of a GFP fusion protein in rice protoplasts. Then the Ubi1301-*PvNHX1* chimeric gene was introduced into the switchgrass genome by *Agrobacterium*-mediated transformation to observe the growth performance and physiological phenotypes under salt stress and provide some insights into new potential functions of NHX1 in plants.

## Materials and methods

### Cloning and bioinformatics analysis

Total RNA was extracted from the leaves of switchgrass cv. Alamo using the TRIzol reagent method (Invitrogen, Carlsbad, CA, USA), and the cDNA was synthesized by a TIANScript RT Kit (Tiangen Biotechnol-ogy Co., Ltd., Beijing, China) with an oligo-dT primer. The cDNA amplification primers (NHX-F1 and NHX-R1) were designed based on multiple known sequences of *NHX1*-like genes from different species (Figure S1). For amplification of full length *PvNHX1* cDNA, rapid amplification of cDNA ends (RACE) was performed by the SMARTTM RACE cDNA Amplification Kit (Clontech, Mountain View, CA, USA). Gene specific primers NHX-P1 and NHX-P2 were designed for amplification of 5′ and 3′ untranslated regions, respectively. These fragments were assembled by overlapping sequences to obtain the full length cDNA of *PvNHX1*. All primers used in this study were listed in Table S1.

The bioinformatics analysis of PvNHX1 were conducted using various bioinformatics tools: the basic local alignment search tool (BLAST) (http://blast.ncbi.nlm.nih.gov/Blast.cgi) was performed on the National Center for Biotechnology Information (NCBI) platform to determine sequence similarity; Multiple sequence alignment was performed for the amino acid sequence alignment using DNAMAN software; Prediction of transmembrane domains were performed with TMHMM Server2.0; Signal peptide prediction was checked using Signal P 4.1 (http://www.cbs.dtu.dk/services/SignalP/); To investigate the phylogenetic relationships of PvNHX1 with other Na^+^ (K^+^)/H^+^ antiporters, phylogenetic analysis was performed with Clustal X and MEGA software using the neighbor-joining method (Tamura et al., 2013).

### Subcellular localization experiments

The open reading frames (ORFs) of *PvNHX1* gene (stop codon removed) was amplified using primers NHX-F2, NHX-R2 (Table S1), and resulting in a sequence with *Bsa* I and *Eco31* I sites. The ORF was inserted between the constitutive CaMV 35S promoter and GFP gene in a pBWA(V)HS-GFP (provided by the BioRun Company) expression vector to generate an in-frame fusion of GFP to the C-terminus of *PvNHX1*. Then, the fusion constructs pBWA(V)HS-*PvNHX1*-GFP was introduced into rice protoplasts prepared from cell culture. As control proteins for tonoplast targeting, we used pBWA(V)HS-GFP and AtTPK1-RFP. For red fluorescence, mKATE, which was a far-red fluorescent protein, fused to AtTPK1 to stain vacuolar membranes. The protoplasts transformation was performed as following the procedure described by Voelker et al. (2006). Visualization of GFP and RFP in the transformed protoplasts were observed by a laser confocal scanning microscope (FV10-ASW, Olympus, Japan) at a wavelength of 488 and 588 nm, respectively.

### Expression analyses of *PvNHX1*

To identify the tissue-specific expression of *PvNHX1* gene, quantitative real-time PCR (qRT-PCR) assay was performed. Total RNA from root, stem and leaf of switchgrass cultivar “Alamo” were isolated for cDNA synthesis using PrimeScriptTM RT reagent kit with gDNA Eraser kit (TaKaRa, Shiga, Japan). The qRT-PCRs were performed using gene specific primers NHX-F3 and NHX-R3 (Table S1). The switchgrass ubiquitin-1 gene (*PvUBQ1*, genbank FL955474.1) was amplified as the internal control with specific primers NHX-F4 and NHX-R4 (Table S1).

We also quantified the development stage- specific expressions of *PvNHX1* gene in leaves (collected and immediately frozen at −80°C until analysis), including the five elongation (E1, E2, E3, E4, and E5) and three reproductive (R1, R2, and R3) stages of switchgrass cultivar “Alamo” as described by Hardin et al. (2013). To assess the effect of salt on the expression pattern of *PvNHX1*, we analyzed the transcript levels of *PvNHX1* exposed to different salt stress (0, 150, 250, and 350 mM NaCl) for different time interval (0, 10, 20, and 30 days) using qRT-PCR.

### Generation and molecular identification of transgenic switchgrass

The ORFs of *PvNHX1* gene was amplified by specific primers NHX-F5 and NHX-R5 (Table S1) and inserted into binary expression vector Ubi1301 (provided by the Sinogene Scientific Company) via the *BamH* I and *Kpn* I sites. The transformation, selection and regeneration of switchgrass cultivar “Alamo” plants were performed with *Agrobacterium*- mediated method as described by Liu et al. (2015). Finally, regenerated plantlets were transferred into soil and cultured in the greenhouse. The plants carrying the empty vector (EV) and wild-type (WT) originated from the same tissue culture process were both taken as controls.

The integration and expression of the transgenes was confirmed by PCR, Southern blot and qRT-PCR analyses. For PCR analysis, genomic DNA of putative transgenic lines and control plants were amplified using primers NHX-F6 and NHX-R6 (Table S1) specific to the expression vector Ubi1301. Southern blot analysis was conducted following the procedure as Liu et al. (2015). In brief, 25 ug of genomic DNA was digested with restriction endonucleases *Kpn* I, separated by 0.8% agarose gel and subsequently transferred to a nylon membrane (GE Healthcare Life Sciences, Indianapolis, IN). Hygromycin phosphotransferase (hyg) gene amplified by specific primers NHX-F7 and NHX-R7 (Table S1) was chosen as the probe. Probe labeling, prehybridization, hybridization and detection were determined according to instructions of the DIG Labeling and Detection starter kit II (Roche Applied Science, Mannheim, Germany). The purified Ubi1301-PvNHX1 plasmid and DNA from a non-transformed plant were used as a positive and negative control, respectively. For gene expression in *PvNHX1* overexpressing transgenic switchgrass plants, leaves were collected from E3 stage of different transgenic lines.

### Phenotypic analysis of transgenic plants

Transgenic and control plants (WT and EV) were transplanted into plastic pots containing a compound medium of soil: vermiculite: humus [1:1:1 (v/v/v)]. Plant height, stem diameter, internode length, tiller number, leaf width and leaf length were recorded. Internode 3 (I3) was used for measuring stem diameter. The leaves of I3 were used to measure leaf blade length and leaf blade width. Ten individual tillers of the same transgenic line were randomly sampled for each parameter measurement.

### Salt-stress tolerance tests

To evaluate salt tolerance, three independent transgenic lines (L1, L3, and L8), with abundant transcripts of PvNHX1 and no obvious phenotypic changes, were selected as representatives and subjected to leaf senescence assay and salinity stress test. Mature leaves (third leaf from the top) from WT, EV and transgenic lines (L1, L3, and L8) were harvested for leaf senescence assay (Jha et al., 2013). Leaves were cut into 4 cm long pieces and floated in NaCl solution with concentration gradients of 0, 150, 250, 350, and 450 mM for 30 days. When the number of tillers were enough for samplings, the uniform tillers of both transgenic and control plants were chosen and trimmed from soil to sand culture for salinity stress test. They were watered with 1/2 × Hoagland nutrient solution supplemented with gradually increasing concentrations of NaCl (0, 150, 250, and 350 mM) every 2 days and continued for 30 days.

### Measurement of growth and physiological parameters

To determine the relative increase in growth difference of the transgenics in salt (150, 250, 350 mM NaCl) relative to control conditions, we measured growth parameters (plant height, leaf width, leaf length, stem diameter) on 0 and 30 days, respectively. The relative increase in growth parameters with and without salt was calculated using the formula: (TG_30_-TG_0_)-(WT_30_-WT_0_).

Leaves of transgenic and control plants were sampled every 10 days for determination of relative water content (RWC), electrolyte leakage (EL), malonaldehyde (MDA), and proline contents. RWC and EL were measured following the description in Bao et al. (2009) and Lutts et al. (1996), respectively; MDA concentration was estimated by the reaction of thiobarbituric acid (TBA) as described by Peever and Higgins (1989); For proline content, the samples were extracted in 3% sulfosalicylic and measured at 520 nm absorbance as described by Bates et al. (1973). In addition, shoots and roots were harvested for Na^+^ and K^+^ detection respectively, using the method described by (Wang et al., 2009).

### Transcript levels analysis of transgenic switchgrass

To determine the molecular mechanism underlying differential phenotype and K^+^ accumulation in the transgenic lines and control plants, we searched for potential cell growth-, flowering-, and potassium transport-related genes in switchgrass genomics resource (https://phytozome.jgi.doe.gov/pz/portal.html#!info?alias=Org_Pvirgatum) (Paudel et al., 2016) using the rice (*Oryza sativa* L.) proteins as a BLAST query. Three cell growth-related (*CNR2, CNR8, FTsZ*), flowering-related (*FLP3, MADS15, MADS6*) and potassium transport-related (*HKT4, HAK5, HAK27*) genes were isolated from switchgrass. Moreover, three flowering-related genes (*FT1, APL1, SL1*) which have been functionally identified as key flowering regulators in switchgrass (Niu et al., 2016), were also chosen for gene expression analyses. Leaves from control plants (WT and EV) and three independent transgenic lines (L1, L3, and L8) were collected for analysis. *PvUBQ1* was used for internal control. All primers used were listed in Table S1.

### Statistical analysis

All experiments were independently performed three times and values were presented as the mean ± SE. Results were subjected to analysis of variance (ANOVA) using the SPSS software (Version 18.0, IBM, Armonk, NY, USA). Significance was defined as *P* <0.05 or *P* <0.01. Figures were created using SigmaPlot version 10 software (Systat Software, Point Richmond, CA, USA).

## Results

### Cloning and bioinformatics analysis of *PvNHX1*

A putative vacuolar Na^+^ (K^+^)/H^+^ antiporter gene was isolated from switchgrass using homologous cloning and RACE method. The full-length cDNA was 1669-bp, which contained a complete open reading frame (ORF) of 1611-bp nucleotides coding for a polypeptide of 536 amino acids protein. The sequence data of *PvNHX1* have been firstly submitted to the GenBank database (accession number: KJ739865). The deduced protein contains 12 putative hydrophobic peak domains and 10 strong transmembrane domains (Figures S2A,B). In the secondary structure, the percentage of alpha helix, extended strand, and random coil were 29, 26, and 45%, respectively (Figure S2C). Moreover, PvNHX1 protein harbors 5 completely conserved domains: NhaP, Na-H-Exchanger, b-cpa1, COG3263, and PRK05326 (Figure S2D). The phylogenetic analysis results showed that PvNHX1 was most closely related to proteins from *Zoysia japomca* and *Oryza sativa* (Figure S3).

### Subcellular localization of PvNHX1

**Figure 1:**
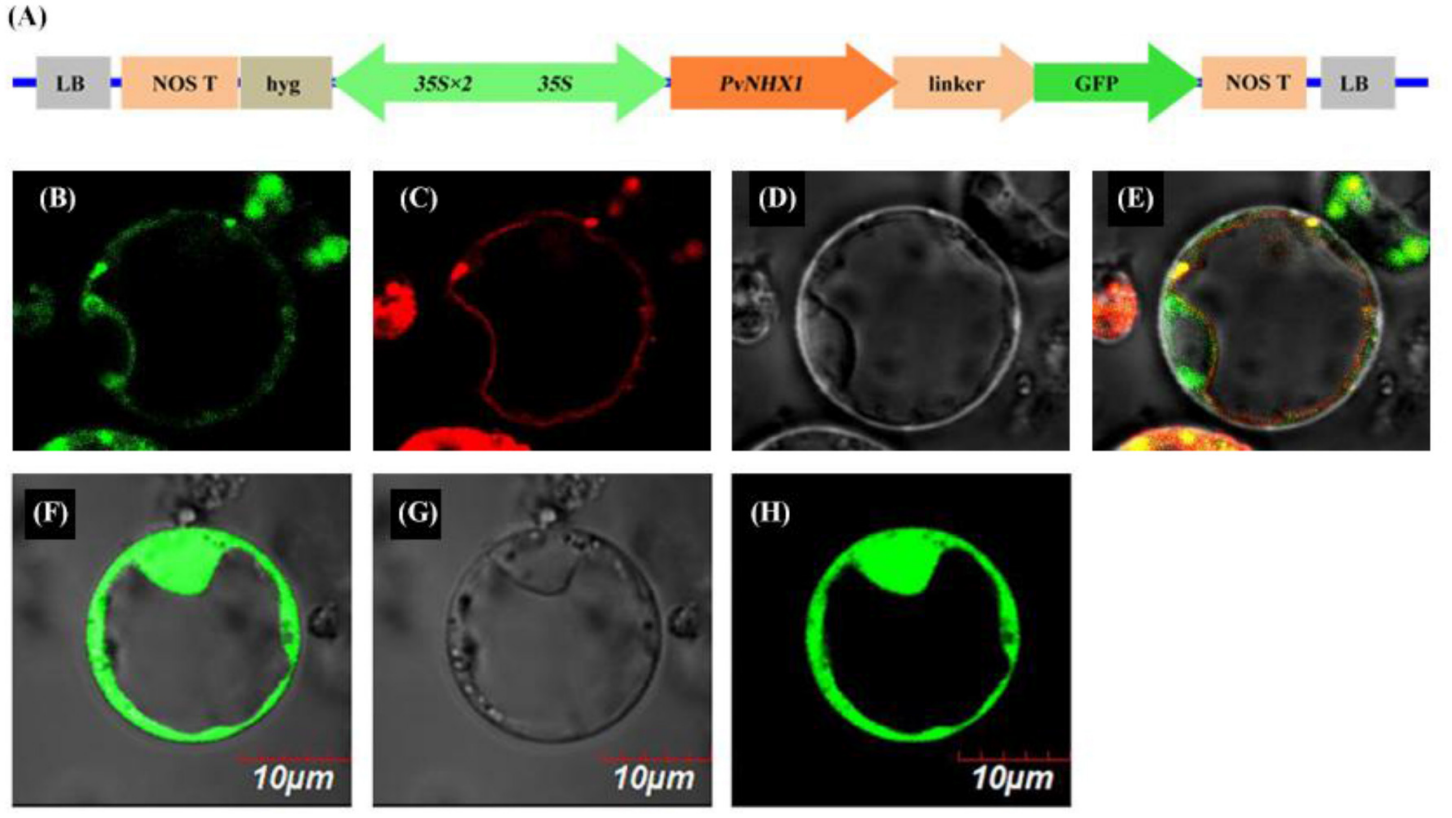
**Subcellular localization of PvNHX1 protein. (A)** Diagram depicting the PvNHX1-GFP fusion construct in vector pBWA (V)HS. *CaMV35S*: cauliflower mosaic virus promoter; Green fluorescence of p35S::PvNHX1-GFP **(B)** and p35S::GFP **(F)** detected in rice protoplasts; **(C)** AtTPK1-mKATE fusion proteins co-localized in the vacuolar membrane; Bright-field image of p35S::PvNHX1-GFP **(D)** and p35S::GFP **(G)**; **(E)** Image of the overlay of the **(B–D)**; **(H)** Image of the overlay of **(F,G)**.

To determine the subcellular location, a *PvNHX1*-GFP fusion protein was constructed under the control of the constitutive CaMV-35S promoter (Figure 1A). In the transient expression of the fusion protein in rice protoplasts, Green fluorescence was visible throughout the cytoplasm in protoplasts with control plasmid p35S::GFP (Figures 1F–H), and red fluorescence was visible predominantly along the vacuole membrane (Figure 1C). In protoplasts with p35S::*PvNHX1*-GFP, green fluorescence (Figure 1B) was consistent with the red fluorescence (Figures 1D,E), indicating the localization of PvNHX1 on the vacuole membrane. Subcellular localization of PvNHX1 was consistent with the phylogenetic analysis.

### Expression patterns of *PvNHX1*

**Figure 2:**
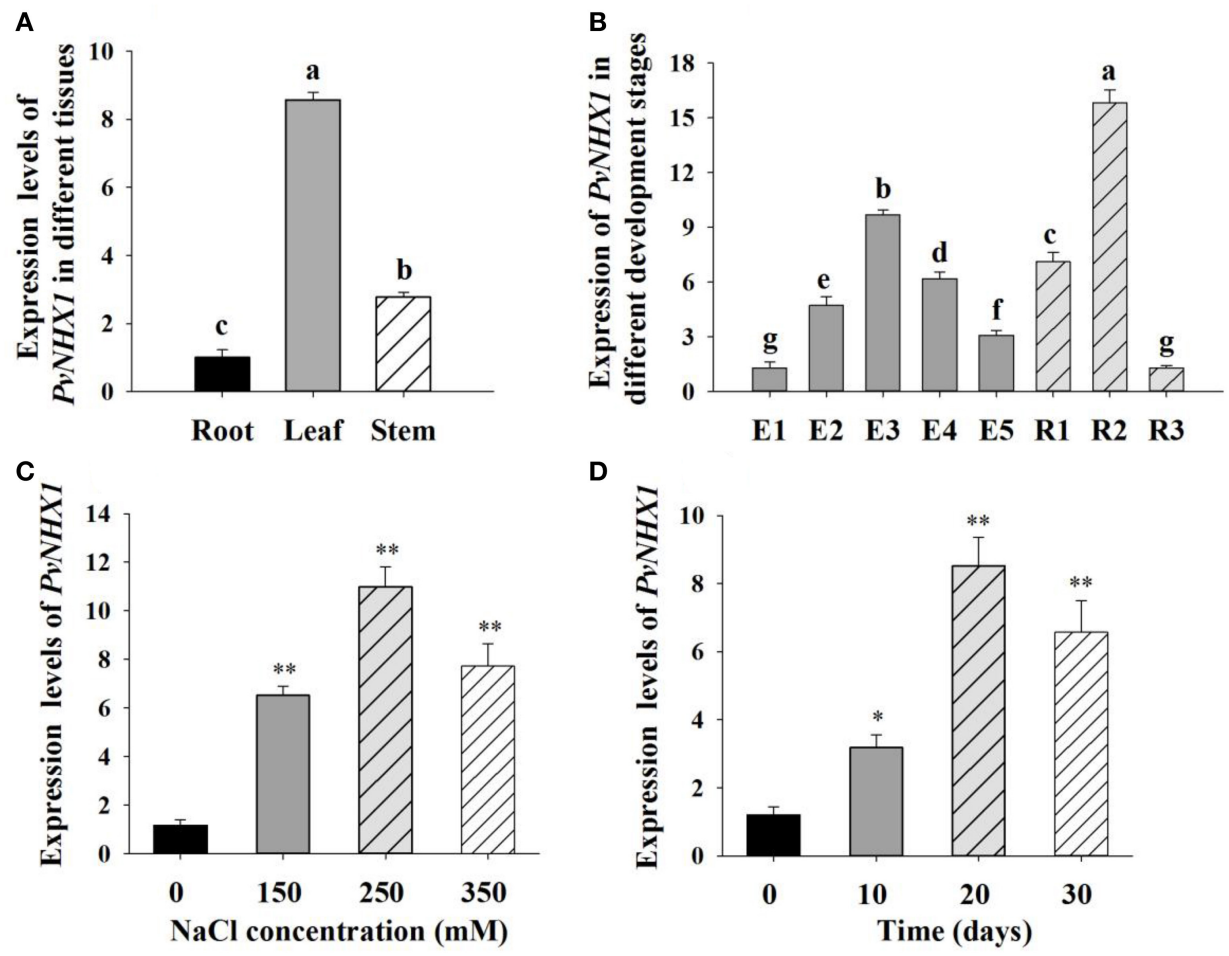
**Expression analysis of *PvNHX1* in switchgrass by qRT-PCR. Relative expression level of *PvNHX1* in different tissues (A)** and different development stages **(B)**; **(C)** Expression of *PvNHX1* under different concentrations of NaCl (0, 150, 250, 350 mM) for 20 days; **(D)** Expression of *PvNHX1* under 250 mM NaCl for 0, 10, 20, and 30 days, respectively. The data represents the mean ± S.E of three replicate samples. ^*^ and ^**^ indicate a significant difference from that of WT at *P* <0.05 and <0.01, respectively. Columns with different letters indicate significant differences at *P* <0.05.

The expression of *PvNHX1* was found in almost all of the tissues in switchgrass. As shown in Figure 2A, relatively higher levels were detected in the leaf and stem which showed 8.57 and 2.78-fold higher (*P* <0.01) compared with that in root, respectively, indicating *PvNHX1* gene preferentially expressed in the leaf tissue.

Developmental stage-specific expression pattern of *PvNHX1*were also analyzed. *PvNHX1* was expressed at a low level in the leaves at the early stages of switchgrass elongation development (E1) and expression started to increase gradually, reaching the maximum value at E3 (9.69-fold than E1, *P* <0.01); then displayed decreasing trends (Figure 2B). For the reproductive stages, the expression of *PvNHX1* exhibited the highest level (15.83-fold) at R2, and start to decline at R3 (1.28-fold).

Significant increase in *PvNHX1* transcript levels was observed in salt treated switchgrass. Under 150, 250, and 350 mM NaCl conditions, the expression levels of *PvNHX1* in leaves increased 6.52- 10.98-, and 7.72-fold, respectively, relative to the control at normal condition (Figure 2C). Analysis of expression levels of *PvNHX1* at various time points following 250 mM NaCl treatment showed 3.17-, 8.52-, and 6.57-fold increase at 10, 20, and 30 days, compared to 0 day, respectively (Figure 2D). Generally, *PvNHX1* gene was induced by NaCl treatment, indicating the potent role of *PvNHX1* in salt tolerance.

### Production and confirmation of transgenic switchgrass

**Figure 3:**
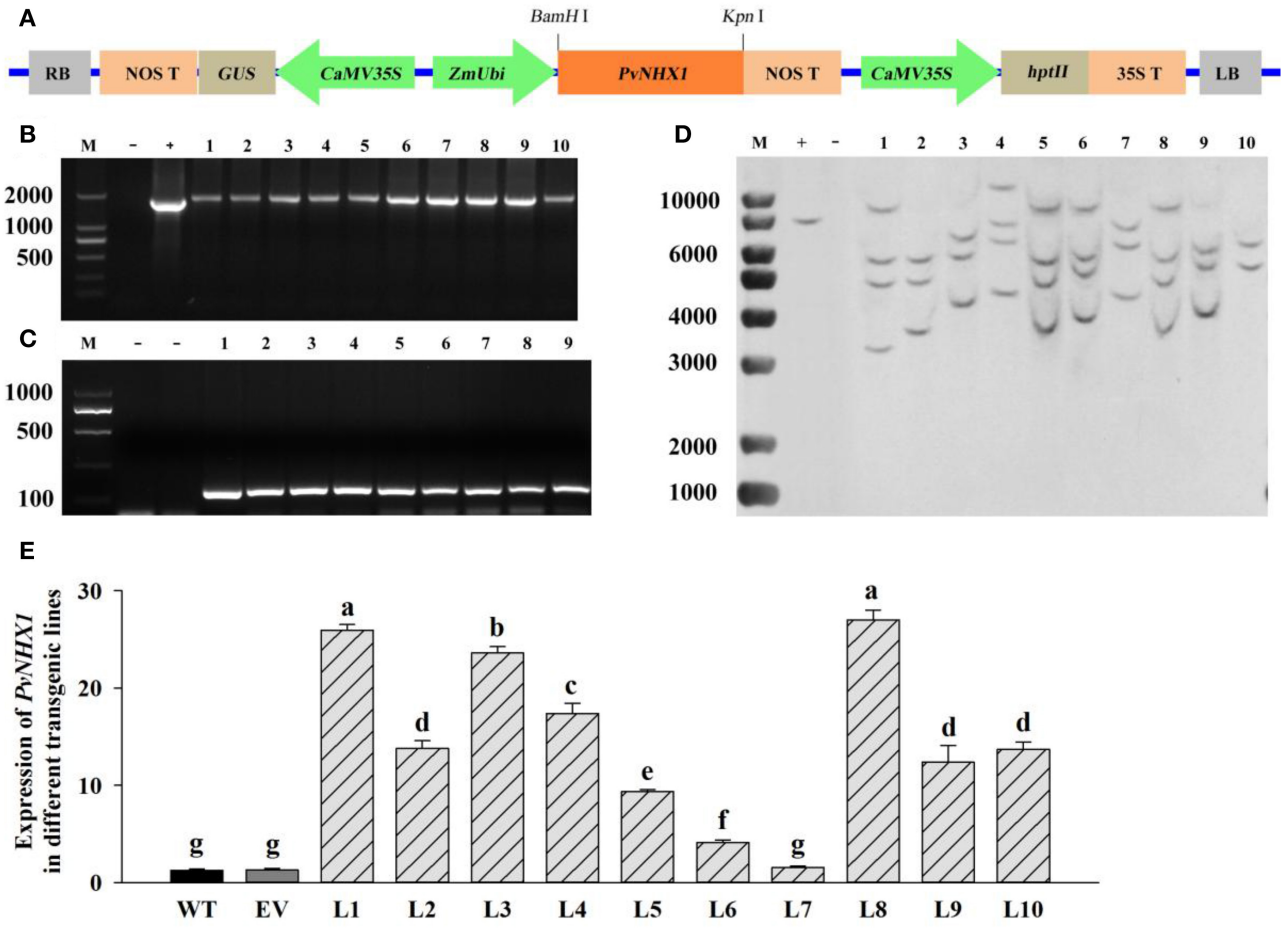
**Molecular analysis of transgenic lines overexpressing *PvNHX1*. (A)** Diagrammatic representation of the overexpression vectors Ubi1301-*PvNHX1*. PCR confirmation of **(B)** *PvNHX1*and **(C)** Ubi1301. M: DL 2000 marker; **(D)**: Southern blot analysis. M: 1 kb plus DNA ladder; +: plasmid (positive control); −: wild type (negative control); **(E)**: Relative expression of *PvNHX1*; Data are presented as mean ± S.E. of triplicate experiments; ^*^ and ^**^ indicate a significant difference from that of WT at *P* <0.05 and *P* <0.01, respectively.

Transgenic switchgrass overexpressing *PvNHX1* was generated by using a binary vectors Ubi1301- *PvNHX1* through *Agrobacterium*-mediated transformation (Figure 3A). The expected sized fragments of 1,800 bp (Figure 3B) and 100 bp (Figure 3C) were amplified in Ubi1301- *PvNHX1* and Ubi1301, respectively. A differential integration pattern of the transgene was observed in transgenic lines; a total of 2–4 copies of *PvNHX1* gene were stably integrated into transgenic lines, whereas no signal was observed in WT (Figure 3D). The occurrence of two or more signals could be due to presence of multiple restriction sites in *PvNHX1*. Among the transgenic lines, L8 exhibited the highest expression, followed by L1 and L3 (Figure 3E).

### Phenotypic analysis of transgenic switchgrass

**Figure 4:**
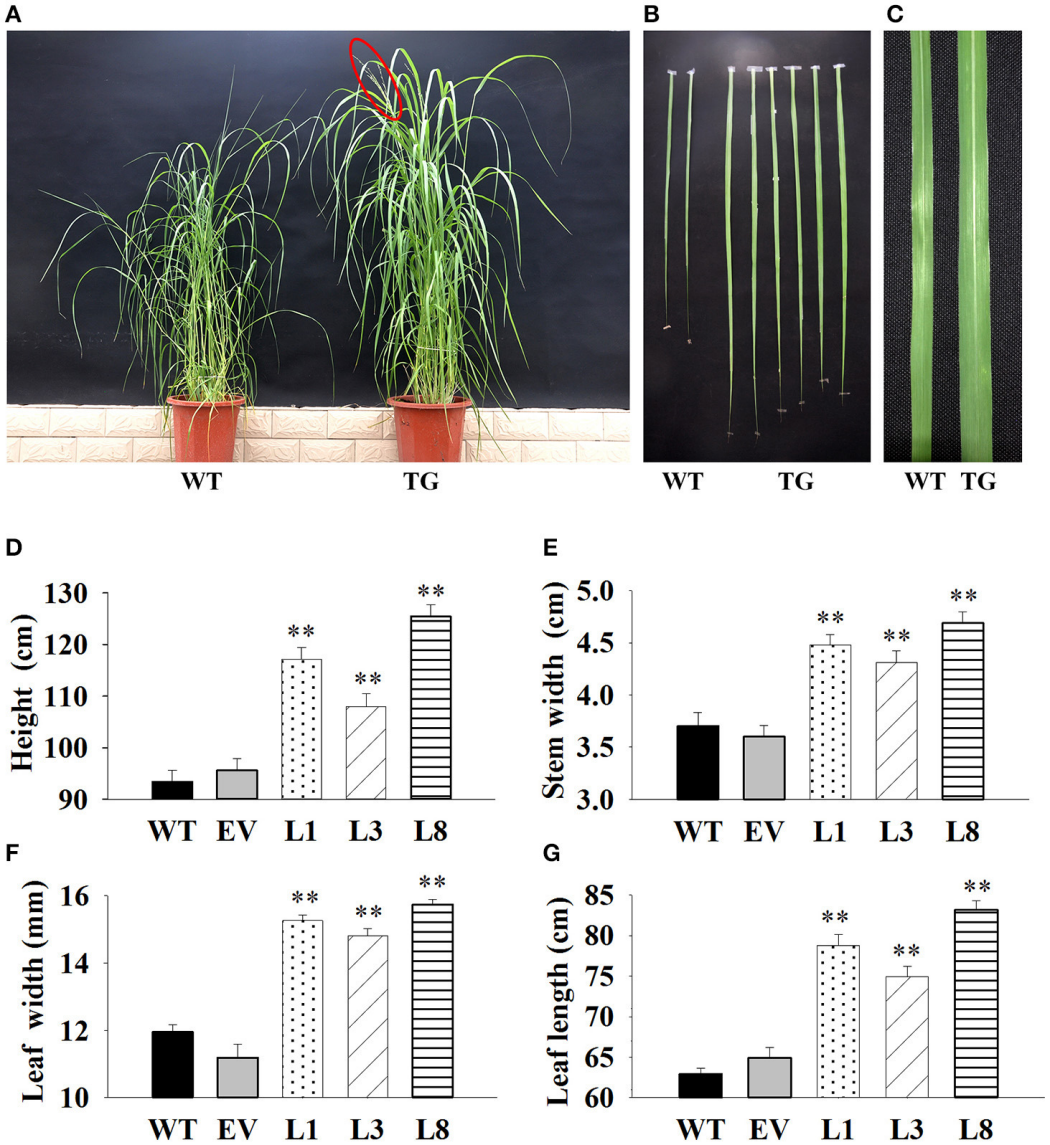
**Phenotypic comparison of transgenic switchgrass under normal conditions. (A):** Phenotypes of whole plants **(A)**, leaf length **(B)** and leaf width **(C)**; Statistical analysis of plant height **(D)**, stem diameter **(E)**, leaf width **(F)** and leaf length **(G)**. WT, wild type; TG, transgenic plants of *PvNHX1*; EV, transgenic lines expressing the empty vector only; L1, L3, and L8: different transgenic lines overexpressing *PvNHX1*. ^*^Indicates significant differences from the WT at *P* <0.05; ^**^indicates significant differences from the WT at *P* <0.01.

We interestedly found that the transgenic lines exhibited better in growth-related phenotypes than control plants under the same greenhouse environment (Figure 4). The vertical height of WT and EV averaged 94.54 cm, whereas that of transgenic plants showed 1.24-, 1.14-, and 1.77-fold increase in L1, L3, and L8, respectively (Figure 4D). The increase of stem diameter in transgenic lines ranged from 18 to 28% (Figure 4E). The transgenic plants also showed an increase by 24 and 32% in leaf width and leaf length, respectively, whereas the corresponding level in control plants averaged only 11.57 and 63.89 cm, respectively (Figures 4F,G).

### Growth adaptation of transgenic switchgrass under salt stress

**Figure 5:**
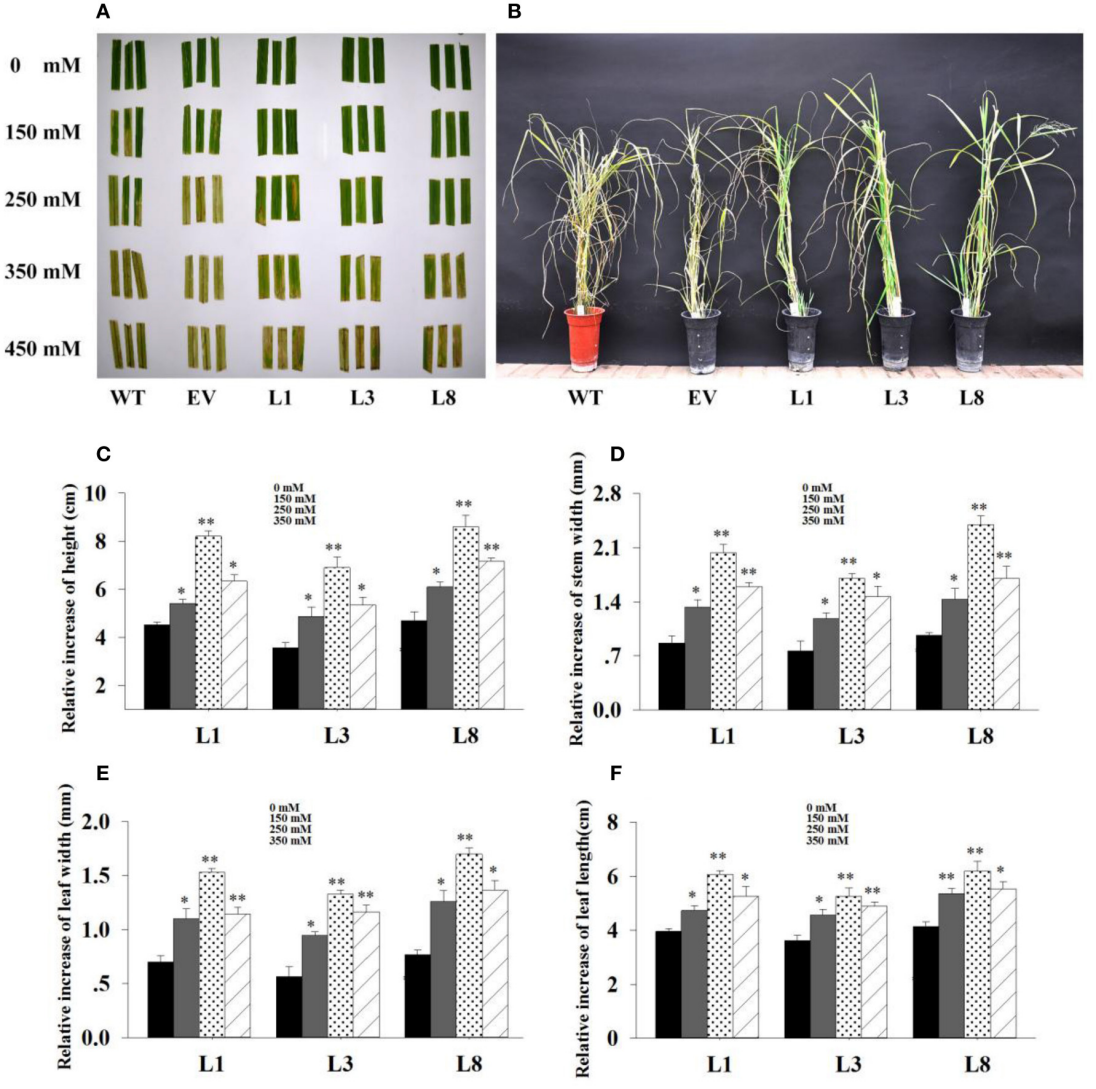
**Phenotype adaptation and relative increase in growth parameters of the transgenics with and without salt. (A)** : Leaf senescence assay under 0, 150, 250, 350 and 450 mM NaCl treatments for 30 days. **(B)**: Phenotypic change of transgenic lines and control plants under 350 mM NaCl treatment for 30 days. Relative increase of plant height **(C)**, stem diameter **(D)**, leaf width **(E)** and leaf length **(F)** of the transgenic lines in salt (150, 250, 350 mM NaCl) relative to control conditions. The data shows the mean ± S.E. of triplicate experiments. ^*^ and ^**^ indicate a significant difference from that under normal conditions at *P* <0.05 and *P* <0.01, respectively.

In leaf senescence assay, the damage caused by salt stress was visualized by the degree of bleaching in leaf tissues. The phenotype of the transgenic and the control plants was similar with normal condition (i.e., 0 mM NaCl); however, the leaves of control plants started to turn yellow at 10 days and exhibited excessive bleaching at 30 days under salt stress. In contrast, the leaves from transgenic lines continuously stayed green even after 30 days of 250 mM NaCl stress (Figure 5A). Similar patterns were also observed on salinity stress test carried out in sand cultures. After grown in normal and low salt conditions (150 mM NaCl) for 30 days, both transgenic and control plants grew vigorously. However, severe growth inhibition, chlorosis and wilting were observed in control plants under high salt conditions; whereas these symptoms were effectively alleviated in transgenic plants, indicating that transgenic plants were more tolerant to salt stress (Figure 5B).

Based on the extra-gain in growth difference of the transgenics in salt relative to control conditions, transgenic lines showed obvious advantages on growth under salt stress. As shown in Figure 5, the relative increase of growth parameters in transgenic lines under salt stress conditions were significantly higher (*P* <0.05 and *P* <0.01) than those under normal conditions. Comparatively, the relative increase of height, stem diameter, leaf width and leaf length in transgenic lines averaged 1.93-, 2.48-, 2.35-, and 1.53-fold higher in salt (250 mM NaCl) relative to normal conditions, respectively (Figures 5C–F). These results implied that overexpression of *PvNHX1* triggered a significant increase in switchgrass growth and biomass under salt stress.

### Physiological responses in transgenic switchgrass under salt stress

**Figure 6:**
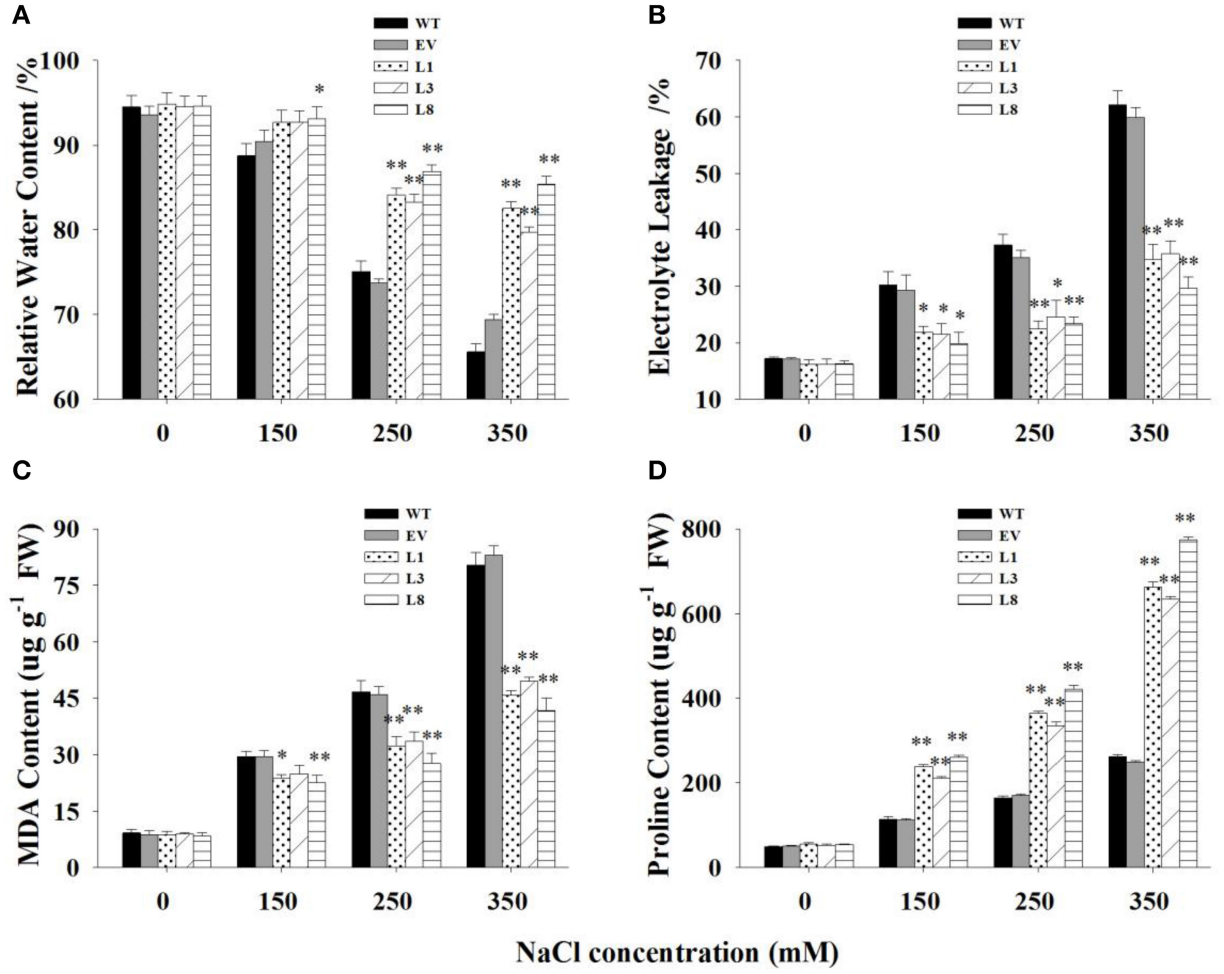
**Physiological analysis of transgenic and control plants under various concentrations of NaCl (0, 150, 250, and 350 mM) treatment for 30 days. Measurement of RWC (A)**, EL **(B)**, MDA **(C)**, and proline content **(D)** in leaves of control plants (EV, WT) and transgenic lines. The data shows the mean ± S.E of three replicate samples. ^*^ Indicates significant differences from the WT at *P* <0.05; ^**^ indicates significant differences from the WT at *P* <0.01.

Transgenic lines and control plants exhibited approximately physiological status regarding RWC, EL, MDA, and proline under normal condition. However, under salt stress, significant differences were observed (Figure 6). Transgenic lines exhibited greater potential to maintain higher tissue water content than control plants. The decrease of RWC in control plants was 21% (*P* <0.01) and 28% (*P* <0.01) respectively under 250 and 350 mM NaCl conditions, whereas that of transgenic lines was only 10 and 13%, respectively (Figure 6A).

EL was measured to examine cell membrane stability under salinity stress. The EL in transgenic lines were significantly lower (*P* <0.01) than control plants under salt stress conditions (Figure 6B). Comparatively, EL of the control plants increased to 117 and 261% under 250 and 350 mM NaCl conditions, respectively, while those of transgenic lines was only 45 and 106%, respectively. Similarly, MDA, an indicator of membrane lipid peroxidation damage, increased gradually with the increase of salt concentrations. The increases in control plants was significantly higher (*P* <0.01) than those in transgenic lines, resulting in 1.68- and 1.99-fold increases under 250 and 350 mM NaCl conditions, respectively (Figure 6C).

With the increase of salt concentration, the proline content increased sharply in all plants but showed a more rapid increase in transgenic lines (Figure 6D). The increase in proline contents in transgenic lines were 2.08-, 2.22-, and 2.70- fold higher than those in control plants under 150, 250, and 350 mM NaCl stress, respectively.

There was no significant difference in these physiological parameters at the treatment beginning (Figure S4). With prolongation of treatment time, obvious differences were observed between transgenic lines and control plants. In general, transgenic lines exhibited significantly higher RWC, reduced malondialdehyde production, preserved cell membrane integrity and increased proline accumulation at every sampling point.

### Transgenic plants accumulated less Na^+^ and more K^+^

**Figure 7:**
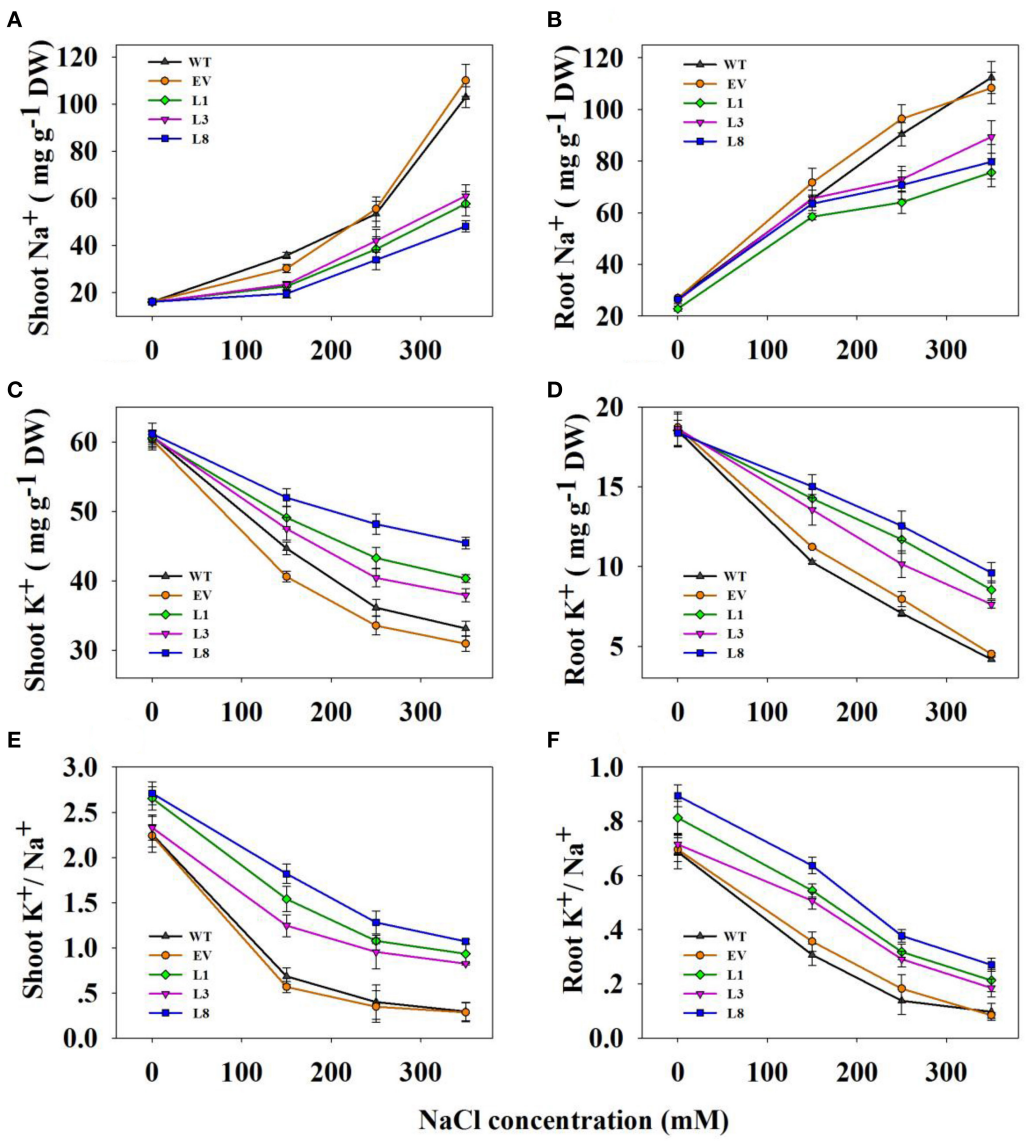
**Na^+^, K^+^ contents and K^+^/Na^+^ ratios in transgenic lines and control plants under different NaCl concentrations (0, 150, 250, and 350 mM) for 30 days. Na^+^ content in shoots (A)** and roots **(B)**; K^+^ content in shoots **(C)** and roots **(D)**; K^+^/Na^+^ in shoots **(E,F)** roots. The data shows the mean ± S.E of three replicate samples.

Transgenic lines and control plants exhibited approximately equal Na^+^ and K^+^ contents as well as K^+^/Na^+^ ratio under normal condition (Figure 7). In transgenic and control plants, salinity treatments led to increased Na^+^ content in all tissues examined, but the increases in control plants were significantly higher than that in transgenic lines (Figures 7A,B). For instance, under 350 mM NaCl stress, control plants accumulated 1.98- and 2.02-fold (*P* <0.01) higher Na^+^ in shoots and roots respectively, compared to transgenic lines. However, high salinity drastically decreased K^+^ concentration in all tissues (Figures 7C,D). Notably, transgenic lines accumulated a higher concentration of K^+^. Compared with control plants, the transgenic lines exhibited 1.24- and 2.06-fold in shoots and roots, respectively under 350 mM NaCl stress.

As a consequence, the ratio of K^+^/Na^+^ decreased significantly when plants were subjected to salinity stress, but higher ratios were observed in the transgenic lines (Figures 7E,F). For instance, the ratio in shoots and roots of the transgenic lines averaged 0.94 and 0.22, respectively, whereas those in the corresponding tissues of control plants were only 0.29 and 0.10, respectively under 350 mM NaCl conditions.

### Transcript levels analysis of transgenic switchgrass

**Figure 8:**
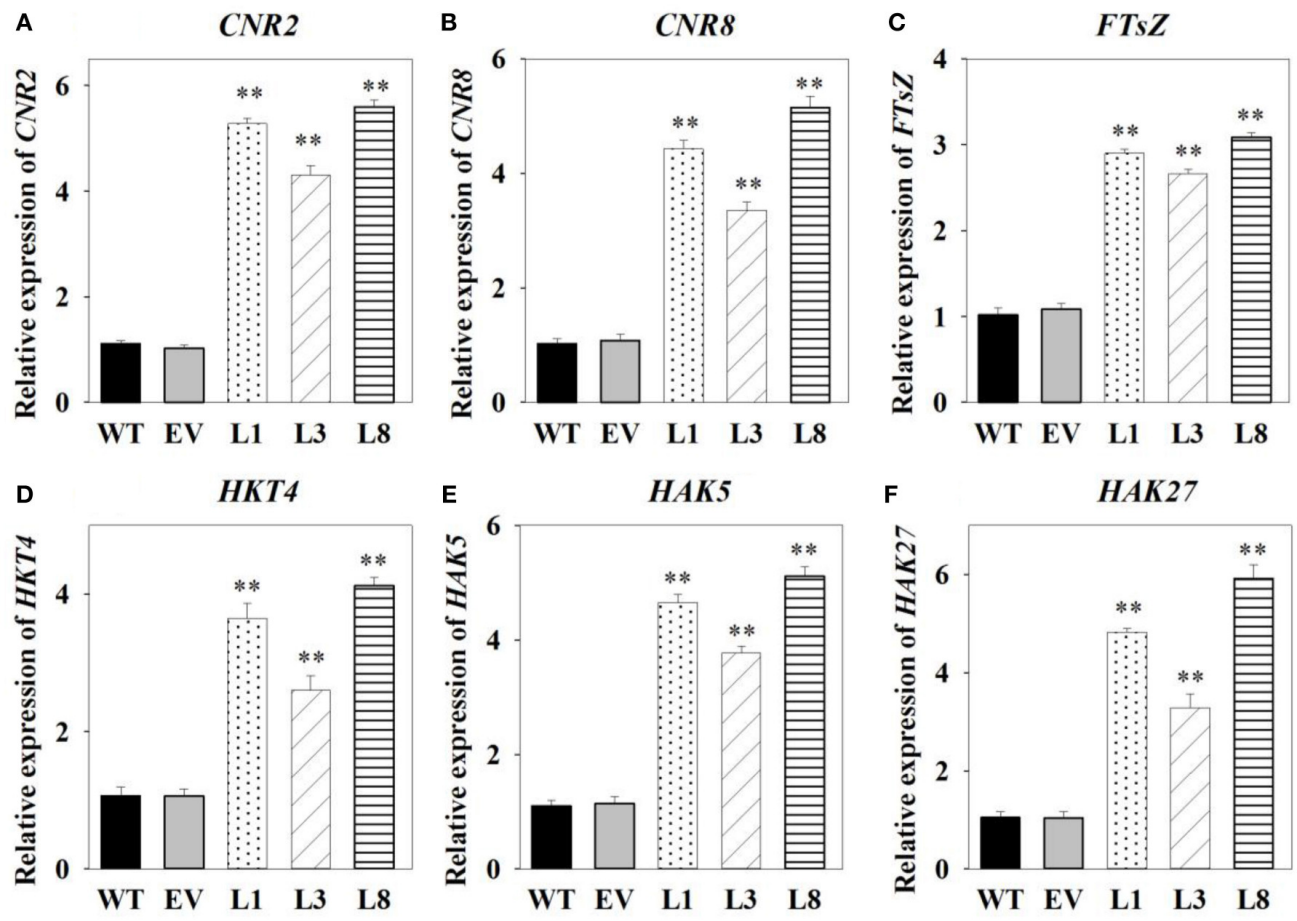
**Expression levels analysis of cell growth-, potassium transport-related genes in transgenic switchgrass**. Expression levels of *CNR2* **(A)**, *CNR8* **(B)**, *FTsZ* **(C)**, *HKT4* **(D)**, *HAK5* **(E)**, and *HAK27* **(F)**. The data shows the mean ± S.E of three replicate samples. ^*^ and ^**^ indicate a significant difference from that of WT at *P* <0.01, respectively.

Having identified genes involved in switchgrass cell growth, flowering and potassium transport, we then analyzed their transcript levels by qRT-PCR in the transgenic and control plants. We found that cell number regulator genes (*CNR2, CNR8*) and cell division gene (*FTsZ*) were significantly up-regulated (*P* <0.01) in transgenic lines, with at least 2.66-fold increase in expression (Figures 8A–C). Transcript levels of three high-affinity potassium transporter genes (*HKT4, HAK5*, and *HAK27*) related to potassium transporters were found to be significantly higher (*P* <0.01) in transgenic lines overexpressing *PvNHX1* than in the control pants (Figures 8D–F). Morever, genes involved in flowering, SUPPRESSION OF OVEREXPORESSION OF CONSTANS1 (SOC1)-like gene (*SL1*), FLOWERING PROMOTING FACTOR 3-like gene (*FLP3*) and MADS-Box gene (*MADS15*) were significantly up-regulated in transgenic lines (*P* <0.01), but expression of FLOWERING LOCUS T1 (*FT1*), APETALA 1 (*AP1*)-like gene (*APL1*), and MADS-Box gene (*MADS6*) decreased at least threefold compared to the control plants (Figures S5).

## Discussion

According to subcellular localizations and physiological roles, Na^+^ (K^+^)/H^+^ antiporters in plants were classified into two families, including plasma membrane transporter (SOS) and intracellular transporter (NHX) (Brett et al., 2005). The NHX family can be further subdivided into two distinct groups, class-I and class-II (Pardo et al., 2006). The class-I NHX proteins were located in the vacuolar membrane and catalyzed Na^+^/H^+^ and K^+^/H^+^ exchanges, whereas the class-II proteins involved in the regulation of K^+^ homeostasis, were localized in various endosomal compartments. According to phylogenetic analysis and subcellular localization, we confirmed that PvNHX1 was a member of the class-I subgroup (Figure S3), that localized to vacuolar membrane in switchgrass (Figure 1). Similar with other NHXs, such as VvNHX1 (Hanana et al., 2007), PvNHX1 protein exhibited 10 transmembrane domains (TM) spanning and contained a conserved amiloride binding sites (LFFIYLLPPI). Additionally, the putative CaM-binding domain, which regulated NHX1 activity at the C-terminus in a Ca2^+^- and pH dependent manner, was found within PvNHX1 protein. This domain in PvNHX1 shared a high similarity with that of AtNHX1. Thus, we suggested that PvNHX1 was a vacuolar Na^+^ (K^+^)/H^+^ antiporter and may have roles in salt stress.

It is intriguing to explore the phenotypes of the transgenic switchgrass overexpressing *PvNHX1* gene. In this study, transgenic switchgrass exhibited better growth and development performance (higher shoot height, larger stem diameter, longer leaf length, and width) than control plants under normal conditions (Figure 4). The growth rate was highly correlated with the expression level of transgenes in different lines, indicating that the increase of growth in transgenic switchgrass directly resulted from the overexpression of *PvNHX1*. Recent reverse genetics evidence has uncovered key regulatory roles and pathways of NHXs that affect plant organ size by altering cell number, cell size, or both (Bassil and Blumwald, 2011; Bassil et al., 2011; Mccubbin et al., 2014). Transcript levels analysis in our study confirmed and strengthened this notion. Cell number regulator genes (*CNR2, CNR8*) (De Franceschi et al., 2013) and cell division gene (*FTsZ*) (Dziadek et al., 2003) were significantly up-regulated (*P* <0.01) in transgenic switchgrass (Figures 8A–C). Besides, *PvNHX1* gene exhibited a developmental-specific expression patterns, and exibited the highest expression levels at R2 stage (Figure 2B), which was defined by fully emerged spikelets with no peduncle present (Hardin et al., 2013). Moreover, strong and stable GUS expression of *NHX* genes were abundant in most flower organs (stigma, pollen, anther, stamen, petal, etc.) of the transgenic rice (Fukuda et al., 2011), *Arabidopsis* (Barragán et al., 2012), and mungbean (Sahoo et al., 2016). These results might suggest that NHXs are involved in plant flower development. This speculation was supported by the reverse genetics evidence in *Arabidopsis*, in which the filaments of *nhx1 nhx2* did not elongate far enough to place anthers and lacked the ability to dehisce and release pollen, resulting in lack of flower set and silique formation (Bassil et al., 2011). More interestingly, experiments in morning glory suggested a close correlation between NHX protein and blue-petal color changes (Yoshida et al., 2009). To gain additional insight into the role of NHXs in plant flowering, we analyzed the transcript levels of the key flowering-time regulators, such as *FT1, APL1, SL1, FLP3, MADS6*, and *MADS15* (Niu et al., 2016). However, transgenic switchgrass overexpressing *PvNHX1* displayed two distinct expression patterns: significantly up-regulation (*SL1, FLP3*, and *MADS15*) and down-regulation (*FT1, APL1*, and *MADS6*) (Figure S5), indicating the role of NHXs in flowering time was complex, not simply to promote or inhibit. Further studies are needed to identify the exact mechanism of NHXs action on plant growth and flowering.

Plant tolerance to salt stress, is well known to be a multigenic trait that involves multiple physiological and biochemical mechanisms and requires the coordinated actions of several genes (Bartels and Sunkar, 2005). However, previous studies in various plant species have demonstrated that overexpressing the single gene, vacuolar Na^+^ (K^+^)/H^+^ antiporter gene, is a feasible way to create transgenic plants with enhanced salt tolerance (Mishra et al., 2014; Chen et al., 2015; Zhang et al., 2015; Sahoo et al., 2016). Consistent with these studies, transgenic switchgrass overexpressing *PvNHX1* exhibited better growth performance (Figure 5) and maintained an improved physiological capacity (Figures 6, Figure S4) comparing to control plants under high salinity stress. However, transgenic switchgrass accumulated significantly higher K^+^ and lower Na^+^ in all tissues compared with control plants (Figure 7), which failed to detect the significant correlation between enhanced salt tolerance and increased accumulation of Na^+^. The presence of lower Na^+^ in transgenic plants overexpressing *NHXs* genes was also reported in rice (Islam et al., 2010), soybean (Li et al., 2010), and cowpea (Mishra et al., 2014), These resules challenged the widely accepted notion that NHXs convey salt tolerance by enhancing Na^+^ sequestration into vacuoles, indicating a different regulatory pathways of NHXs in salt tolerance might exist. This speculation was supported by the evidence from recent reverse genetics, in which the critical role of NHXs in K^+^ homeostasis to withstand salt shock was confirmed (Bassil et al., 2011; Barragán et al., 2012; Andrés et al., 2014). To understand the molecular mechanism underlying the K^+^ accumulation in the transgenic plants, we analyzed the expression levels of three potassium transport-related genes. As expected, K^+^ transporter gene (*HKT*) (Gierth and Mäser, 2007) and K^+^ uptake genes (*HAK5* and *HAK27*) (Horie et al., 2011) were significantly up-regulated in transgenic plants (Figures 8D–F). Our results strongly suggested that NHX proteins mainly functioned as a K^+^/H^+^ antiporter and played an important role in regulating K^+^ homeostasis by regulating their uptake, transport and spatial distribution.

In the present study, we obtained the full-length cDNA of *PvNHX1*, including 5′ UTR, 3′ UTR and ORF. Phylogenetic tree and Subcellular localization analysis confirmed that *PvNHX1* was a vacuolar Na^+^ (K^+^)/H^+^ antiporter belonging to the NHX-I cluster in NHX family. Gene expression analysis revealed *PvNHX1* gene was preferentially expressed in leaves, and highly induced by salt stress. Moreover, our results also confirmed the role of PvNHX1 protein in the regulation of switchgrass growth, development and salt tolerance. To our knowledge, this is the first study that isolated the *PvNHX1* gene from switchgrass and monitored its role on growth and salt tolerance in switchgrass. Our data will provide a valuable foundation for further researches on the potential roles of NHXs in plants.

## Author contributions

Conceived and designed the experiments: YZ and FY. Performed the experiments: YH, CG, YL and XC. Analyzed the data: YH, BC and SY. Wrote the paper: YH and YZ. All authors reviewed and approved the final manuscript.

### Conflict of interest statement

The authors declare that the research was conducted in the absence of any commercial or financial relationships that could be construed as a potential conflict of interest.

## Supplementary material

Figure S1 **The putative conserved sequences of *NHX1*-like genes from different species**. The singly underlined regions represent the primers (NHX-F1 and NHF-R1). Doubly underlined regions represent the highly conserved motifs of *NHX1*.

Figure S2 **Bioinformatics analysis of PvNHX1. (A)** Hydrophobicity plot of PvNHX1; **(B)** Hydrophobicity analysis; **(C)** Secondary structure prediction; **(D)** Conserved domain prediction.

Figure S3 **Phylogenetic analysis of PvNHX1 with related Na^+^ (K^+^)/H^+^ antiporters from different plant species**. Sources of NHXs and their GenBank accession numbers are as follows: PvNHX1 (KJ739865); ZjNHX1 (ABY 193111.2); TaNHX1 (AAS17949.1); SbNHX1 (ACA33931.1); LeNHX2 (CAC83608.1); ZmNHX1, 2 (AAP20428.1), (AAP20429.1); OsNHX1, 2, 3, 5 (BAA83337.1); (AAM08406.1), (AAM08407.1), (BAG92828.1); AtNHX1, 2, 5, 6 (NP_198067.1), (NP_001326210.1), (NP_175839.2), (NP_178079.2); HvNHX1-4 (BAC56698.1), (ANS57040.1), (ABD62853.1), (AAS17948.1); AtSOS1 (NP_178307.2); McSOS1 (ABN04858.1); GmSOS1 (AFD64746.1); PeSOS1 (ABF60872.1); PcSOS1 (BAF41924.1); OsSOS1 (AAW33875.1); SjSOS1 (BAE95196.1).

Figure S4 **Physiological analyses of control and transgenic lines treated with 350 mM NaCl solution for 0, 10, 20, and 30 d, respectively**. The variation tendency of RWC **(A)**, EL **(B)**, MDA **(C)**, and proline content **(D)** under different treatment time; The data shows the mean ± S.E of three plicate samples.

Figure S5 **Expression levels analysis of flowering-related genes in transgenic switchgrass**. Expression levels of *SL1* **(A)**, *FLP3* **(B)**, *MADS15* **(C)**, *FT1***(D)**, *APL1* **(E)**, and *MADS6* **(F)**. The data shows the mean ± S.E of three replicate samples. ^*^ and ^**^indicate a significant difference from that of WT at *P* <0.05 and *P* <0.01, respectively.

Table S1 **Primer sequences used in the experiments**.
