# Supplementary material for: Enhanced Growth Performance and Salinity Tolerance in Transgenic Switchgrass via Overexpressing Vacuolar Na+ (K+)/H+ Antiporter Gene (PvNHX1)
Source: Front Plant Sci. 2017 Apr 3;8:458. doi: 10.3389/fpls.2017.00458 (PMC5376569; doi:10.3389/fpls.2017.00458)

**Table S1** Primer sequences used in the experiments

| **Primer name** | **Primer sequence (5'-3')** |
| --- | --- |
| NHX-F1 | CGTGGTGGCGCACCTGGT |
| NHX-R1 | CTGCTCGGTGGGTGAT |
| NHX-F2 | CAGTggtctcACAACATGGGGCTCGGCTTGGTGGC |
| NHX-R2 | CAGTggtctcATACACCGTCCACTATGCTCGGTGG |
| NHX-P1 | TGGTCGGAGGTGGAGCCCAGCACGCCC |
| NHX-P2 | TCACCAGCACAATCACTGTCGTCCT |
| NHX-F3 | TTGTGCTCTTCAATGCGCTT |
| NHX-R3 | GCAATCCAGCAAACACTCCA |
| NHX-F4 | CAGCGAGGGCTCAATAATTCCA |
| NHX-R4 | TCTGGCGGACTACAATATCCA |
| NHX-F5 | ATGGGGCTCGGCGTGGTGGC |
| NHX-R5 | TCACCGTCCACTCTGCTCGGTGGGT |
| NHX-F6 | TGATGGCCCTGCCTTCATACGCT |
| NHX-R6 | TATTGCCAAATGTTTGAACG |
| NHX-F7 | ATTTGTGTACGCCCGACAGT |
| NHX-R7 | GGATATGTCCTGCGGGTAA A |
| *CNR2*-F | GTGCGTCTTCTCCTGCTTCT |
| *CNR2*-R | CTTCTTGAGCTCGCGGTACT |
| *CNR8*-F | GCTGTTTGCCTTACACTGGTC |
| *CNR8*-R | ATTGTAGCGCCGACGAATAG |
| *FTsZ*-F | GTTGAGGTTGTGGACCCATT |
| *FTsZ*-R | CCACCTCCAACTCCAACAAC |
| *HKT4*-F | TCAGCATTTCTGCCAACAAC |
| *HKT4*-R | GGCCAAGATAGTGCGAGAAC |
| *HAK5*-F | AATCGTACCAAGAGGGCTCA |
| *HAK5*--R | ACGCCATCACCAATTACCAT |
| *HAK27*-F | TCTCCCTTGGTGGCATACTC |
| *HAK27*--R | TGATGGGACTAAACCGAAGC |
| *FT1*-F | CTCAGGGAGTACTTGCACTG |
| *FT1*-R | CTCAGGGAGTACTTGCACTG |
| *APL1*-F | TCCACAAAACATCTGCTACCC |
| *APL1*-R | GATGCGAAGTTGCGGTTG |
| *SL1*-F | GAAGGAGAAGGAGATGACACTG |
| *SL1*-R | GACTTCTGCAACCACGTTTC |
| *FLP3*-F | AGCGCTACGTGAACGAC |
| *FLP3*-R | GAGCCTGAACCTGGCGAA |
| *MADS15*-F | AATTGGTGCCATGAATACAGGA |
| *MADS15*-R | TCAGAGACTCTAGATCCTCTCCCA |
| *MADS6*-F | AGCCACGACAAGCTACGAGT |
| *MADS6*-R | CTGGAAACATTGCTCCGTCT |

Fig. S1


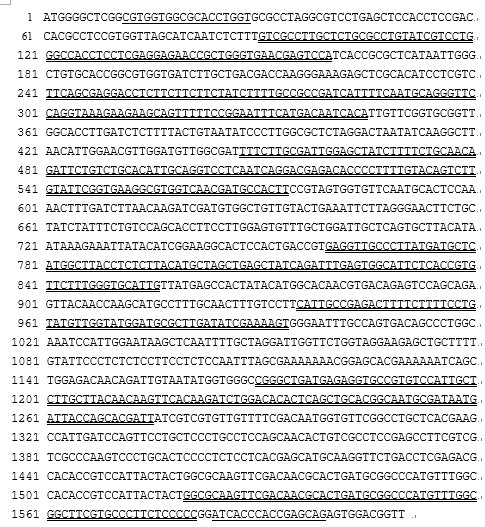


Fig. S2


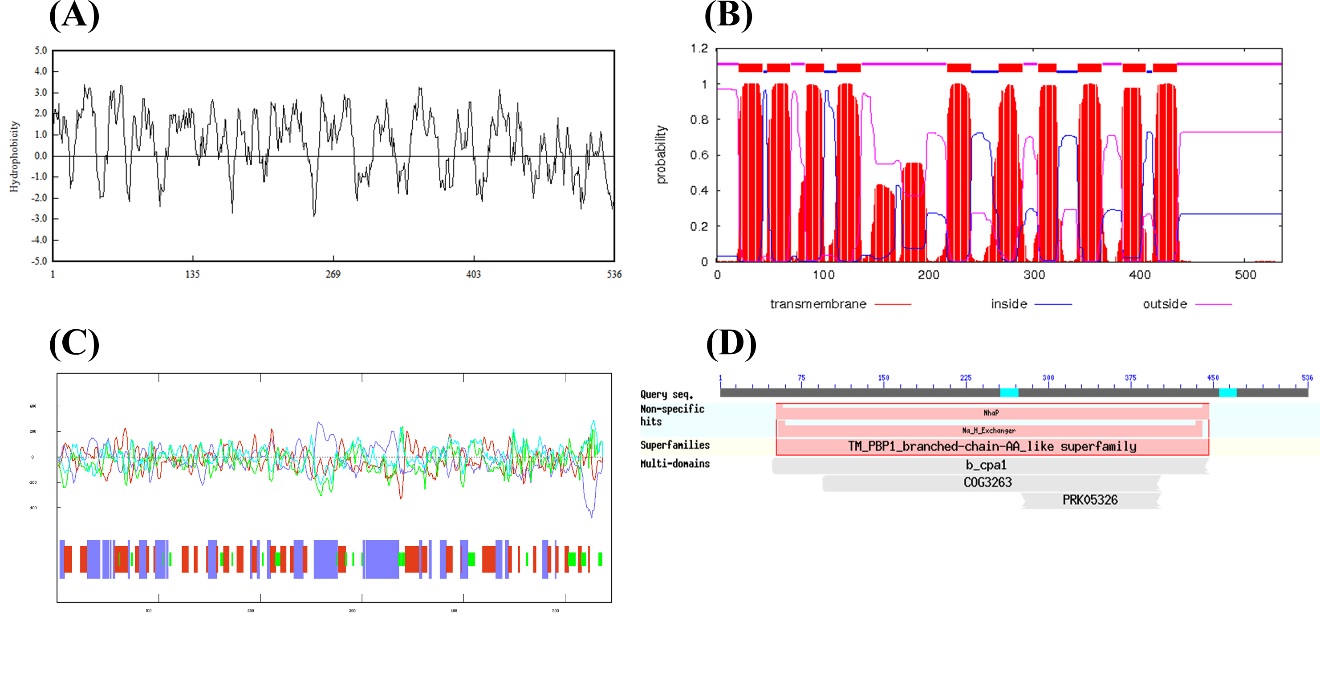


Fig. S3


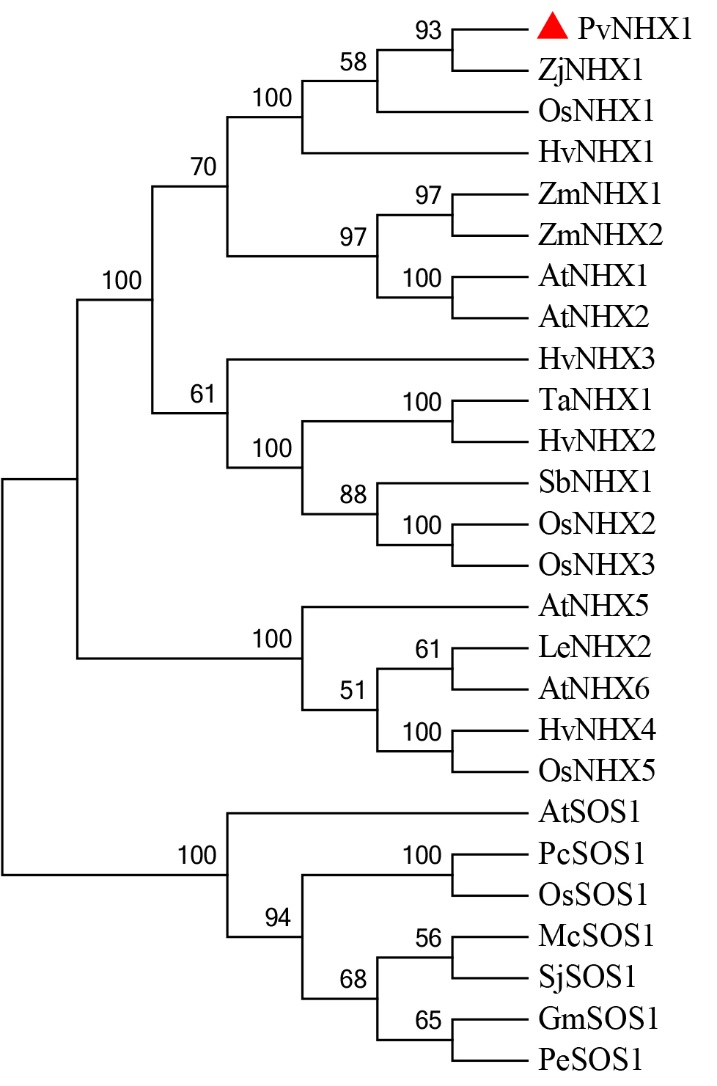


Fig. S4


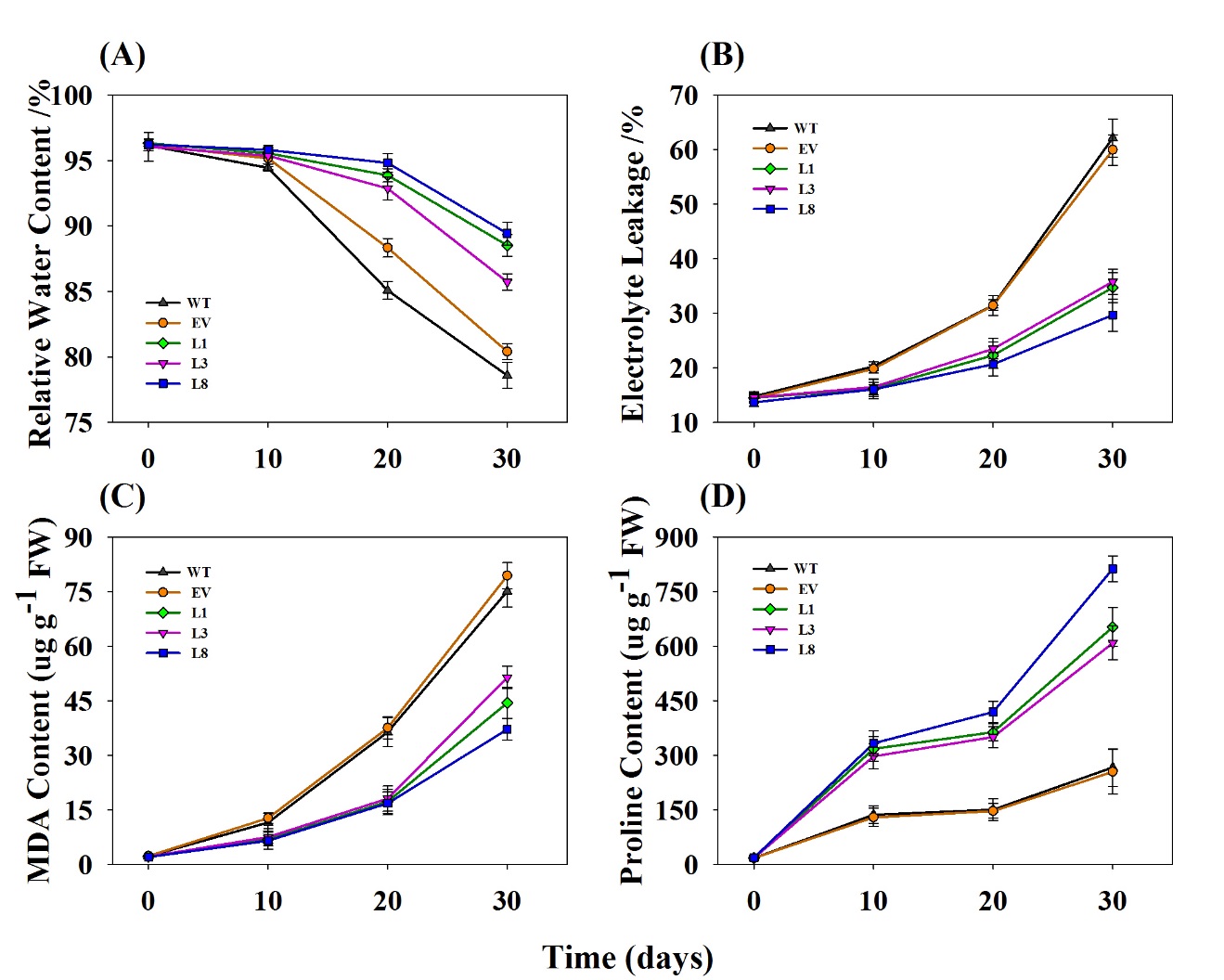


Fig. S5


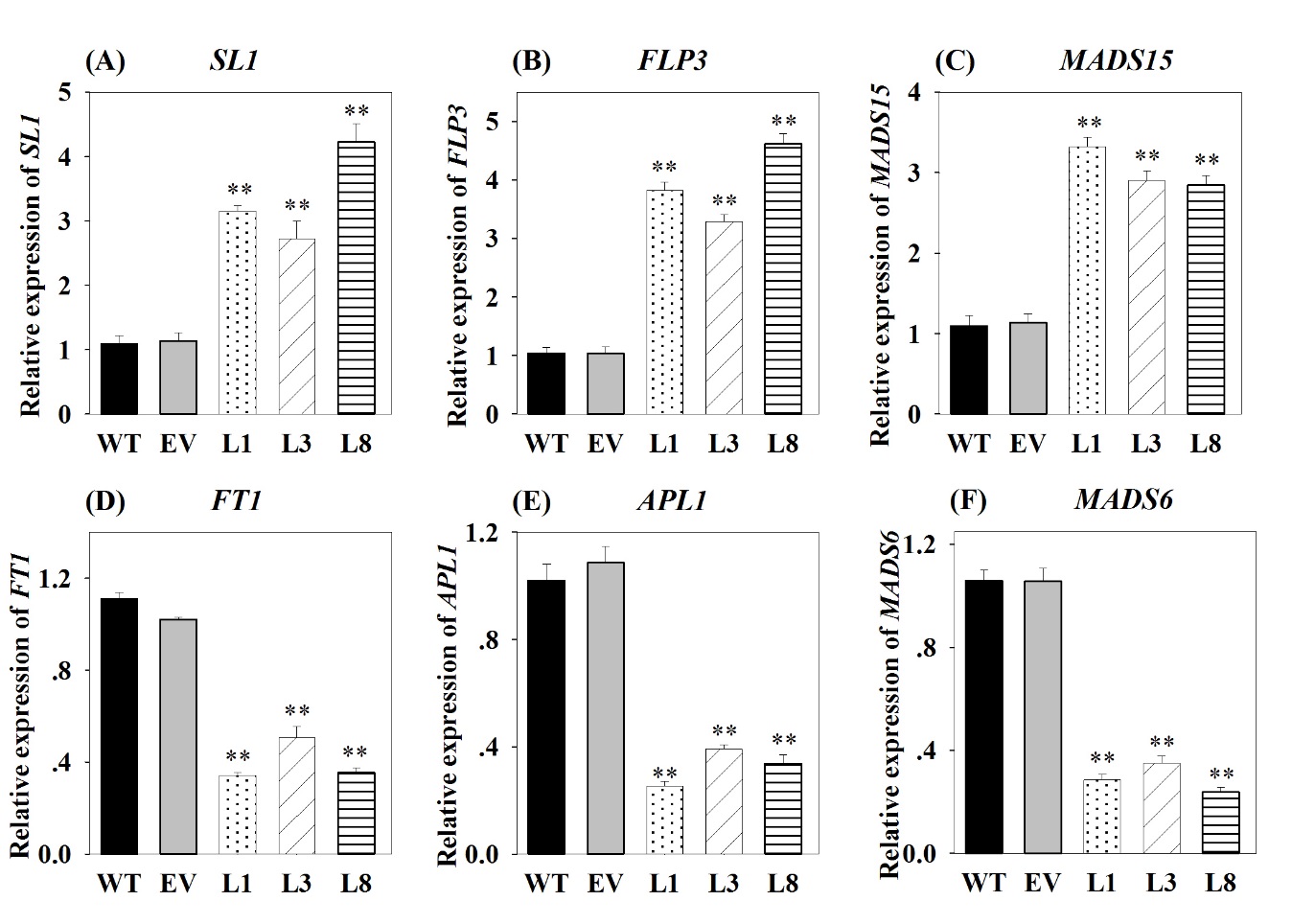

Supplement: Figure S1 — The putative conserved sequences of NHX1-like genes from different species. The singly underlined regions represent the primers (NHX-F1 and NHF-R1). Doubly underlined regions represent the highly conserved motifs of NHX1. [file DataSheet1.docx]
